# Characterization of ribosome heterogeneity during endothelial to hematopoietic transition

**DOI:** 10.1002/2211-5463.70078

**Published:** 2025-06-30

**Authors:** Xitong Tian, Di Liu, Bing Liu, Yu Lan, Jie Zhou

**Affiliations:** ^1^ State Key Laboratory of Experimental Hematology, Haihe Laboratory of Cell Ecosystem, Senior Department of Hematology, Fifth Medical Center Chinese PLA General Hospital Beijing China; ^2^ Department of Neurology, Xuanwu Hospital Capital Medical University National Center for Neurological Disorders Beijing China; ^3^ State Key Laboratory of Experimental Hematology, Haihe Laboratory of Cell Ecosystem Key Laboratory for Regenerative Medicine of Ministry of Education, Institute of Hematology and Blood Diseases Hospital Chinese Academy of Medical Sciences and Peking Union Medical College Tianjin China

**Keywords:** definitive hematopoiesis, endothelial to hematopoietic transition, mRNA translation, ribosome heterogeneity

## Abstract

Emerging evidence has demonstrated that ribosomes are not homogeneous structures with non‐specialized functions. There are now several reports of heterogeneity in the composition and functional specialization of ribosomes. Ribosome heterogeneity functions in regulating the translation of specific mRNAs, and thus plays important roles in embryonic development. However, the panorama of ribosome heterogeneity during embryonic hematopoiesis has not yet been portrayed. Here, by leveraging our single‐cell transcriptomic data and a published proteomic dataset, we depict the landscape of ribosomal heterogeneity during endothelial‐to‐hematopoietic transition (EHT). By precisely distinguishing the different ribosomal components, we found their number and expression levels showed dynamic changes during EHT. We also report stage‐specific signatures of ribosomal components. For example, RPL27 and RACK1 exhibited up‐regulated expression both in dual‐omics analysis and immunofluorescence experiments during EHT. Interestingly, further spatial structure analysis revealed that RACK1 localized at the bottom of 40S small ribosomal subunit, indicating its potential role in regulating ribosome function. Taken together, our study not only highlights the ribosome heterogeneity during EHT, but also provides new clues to explore how these heterogeneous machineries regulate mRNA translation.

## Graphical abstract

The panorama of ribosome heterogeneity during embryonic hematopoiesis has not yet been portrayed. In this study, utilizing dual‐omics data, the heterogenous dynamic of ribosome during endothelial‐to‐hematopoietic transition has been systemically described. Moreover, stage‐specific upregulation and peripheral localization of RPL27 and RACK1 in hemogenic endothelial cells further delineates their specific role in regulating ribosome functions.

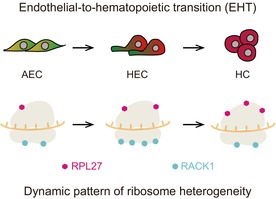

**Abbreviations**
AEC: aortic endothelial cell
EC: endothelial cell
EHT: endothelial‐to‐hematopoietic transition
HC: hematopoietic cell
HEC: hemogenic endothelial cell
HSC: hematopoietic stem cell
PDB: Protein Data Bank
pre‐HSC: pre‐hematopoietic stem cell
RAP: ribosome‐associated protein
RP: ribosomal protein
RPL: large ribosomal subunit protein
RPS: small ribosomal subunit protein

As a macromolecular complex, the mammalian ribosome is composed of four ribosomal RNAs and 80 core ribosomal proteins (RPs), and responsible for uniform mRNA translation [1]. The traditional view holds that ribosomes are homogeneous structures with non‐specialized regulatory function. However, the component homogeneity and functional simplicity of ribosomes have always been questioned.

Because of the large complexity of composition, it is not hard to understand that the ribosome heterogeneity can be manifested in multiple aspects [2]. The contribution of ribosome biogenesis leads to the heterogeneity of ribosomes, mainly reflected in (a) ribosomal RNA variants caused by transcription, processing and modification [3] and (b) cellular specific expression, post‐translational modification, differential stoichiometry and components of RPs and paralogs [2, 4]. In addition, more than 200 ribosome‐associated proteins (RAPs) are also involved in the assembly and maturation of ribosomes, the specificity of which ultimately determine the structure of ribosomes and even their localization in cells [5, 6].

Recently, combining of single‐cell transcriptomics, high‐quality proteomics and ribosome‐profiling technologies, the heterogeneity and regulatory specificity of ribosomes have been gradually excavated in various developmental biological processes [7]. This is mainly because different RPs, such as large ribosomal subunit protein (RPL) and small ribosomal subunit protein (RPS), have different affinities for recognizing the internal ribosome entry sites in the untranslated regions of specific mRNAs, thus regulating the translation efficiency [2]. For example, RPL10A/uL1‐containing ribosomes tend toward the translation of Wnt pathway related genes that control paraxial mesoderm lineage formation during embryogenesis [8, 9]. RPL38‐containing ribosomes tend toward to the translation of Hox family mRNA subgroups, and RPL38 mutations could lead to a short tail homologous alien transformation phenotype [10]. Most importantly, cellular ‘specialized ribosomes’ are also identified. For example, a class of ‘immunoribosome’ can be rapidly formed in response to inflammatory stimuli, which plays a specific immune regulatory function [11].

In mice embryogenesis, definitive hematopoiesis stage starts from the embryonic day 10.0. Though endothelial to hematopoietic transition (EHT), some aortic endothelial cells (AECs) specialize into hemogenic endothelial cells (HECs), then go through two types of pre‐hematopoietic stem cells (HSCs) (T1 and T2 pre‐HSC), and finally develop into functional HSCs [12, 13, 14, 15, 16, 17, 18, 19]. During this process, cell fate and morphology change dramatically [20, 21]. Importantly, our recent work first revealed the important function of ribosome biogenesis and its underlying regulatory mechanism of ribosome biogenesis in regulating EHT [22]. However, the panorama of ribosome heterogeneity during embryonic hematopoiesis has not yet been portrayed. In the present study, utilizing single‐cell transcriptome and bulk proteome data, we fully describe the heterogenous dynamics of ribosome during EHT, and pave a way to understanding the mechanism of ribosome heterogeneity in regulating embryonic definitive hematopoiesis.

## Materials and methods

### Dataset source

The single‐cell transcriptome sequencing data were downloaded from GEO NCBI's Gene Expression Omnibus (GEO) with accession numbers GSE185555 and GSE67120 [22]. The proteomics data, including the acquired raw files, were downloaded from the ProteomeXchange Consortium with the dataset identifier PXD042649 and via the MassIVE partner repository with the dataset identifier MSV000092085 [23].

### Identification of stage‐specific genes/proteins

To identify stage‐specific genes associated with ribosomal biogenesis across different cell populations, we utilized the FindAllMarkers function in Seurat (https://satijalab.org/seurat/), comparing each stage to the others using the Wilcoxon rank sum test. The analysis was conducted using predefined ribosomal biogenesis‐related gene sets [22], including genes involved in RPL, RPS and RAP. Genes with a fold change > 1.5 and *P* < 0.05 were considered significantly differentially expressed.

Stage‐specific proteins were defined as the ratio of protein expression in a given stage to the average expression across the remaining two stages, with the ratio exceeding 2.

### Differential gene expression analysis

After data preprocessing, gene expression between the two populations was compared, and differentially expressed genes were identified using the Wilcoxon rank‐sum test. Genes with a fold change > 1.5 and *P* < 0.05 were considered significantly differentially expressed.

### Immunofluorescence experiment

AECs (CD41^−^ CD43^−^ CD45^−^ CD31^+^ CD201^−^ Kit^−^ CD44^+^), HECs (CD41^−^CD43^−^ CD45^−^ CD31^+^ CD201^+^ Kit^+^ CD44^+^) and hematopoietic cells (HCs) (CD45^+^ Kit^+^ hematopoietic cells) were sorted by flow cytometry, seeded into 24‐well plates pre‐coated with poly‐l‐lysine (Sigma‐Aldrich, St Louis, MO, USA) and incubated overnight. Subsequently, cells were fixed with 4% paraformaldehyde for 30 min at room temperature, followed by permeabilization with 0.1% Triton X‐100 for 10 min. Non‐specific binding sites were blocked with 5% goat serum (Zhongshan Golden Bridge, Beijing, China) at room temperature for 30 min. Primary antibodies were applied and incubated at 4 °C overnight. After washing three times with phosphate‐buffered saline, cells were incubated with species‐matched secondary antibodies for 30 min at room temperature. After three washes in phosphate‐buffered saline, cells were stained with DendronFluor TSA (Histova, Beijing, China; dilution 1 : 100; 60 s). The antigen–antibody complexes were eluted using AbCracker Elution Buffer (Histova) at 37 °C for 30 min to enable sequential labeling of subsequent primary antibodies. After all the antibodies were detected sequentially, the cells were finally stained with 4´,6‐diamidino‐2‐phenylindole. Fluorescence images were acquired using a laser scanning confocal microscope (LSM 980; Zeiss, Oberkochen, Germany) with consistent acquisition parameters across samples. The primary antibodies were as follows: RACK1 (Proteintech, Rosemont, IL, USA; dilution 1 : 200), RPL27 (Proteintech; dilution 1 : 50), RPS15a (Boster, Pleasanton, CA, USA; dilution 1 : 50) and RPS6 (Proteintech; dilution 1 : 50).

### Quaternary ribosome structures

Crystal structures from mouse ribosomes were downloaded from the Protein Data Bank (PDB) (7cpu). All structures were generated using chimerax, version 1.9 (https://www.cgl.ucsf.edu/chimerax). Labels were manually added.

## Results

### The dynamic changes of ribosomal genes during EHT at the transcriptome level

**Fig. 1:**
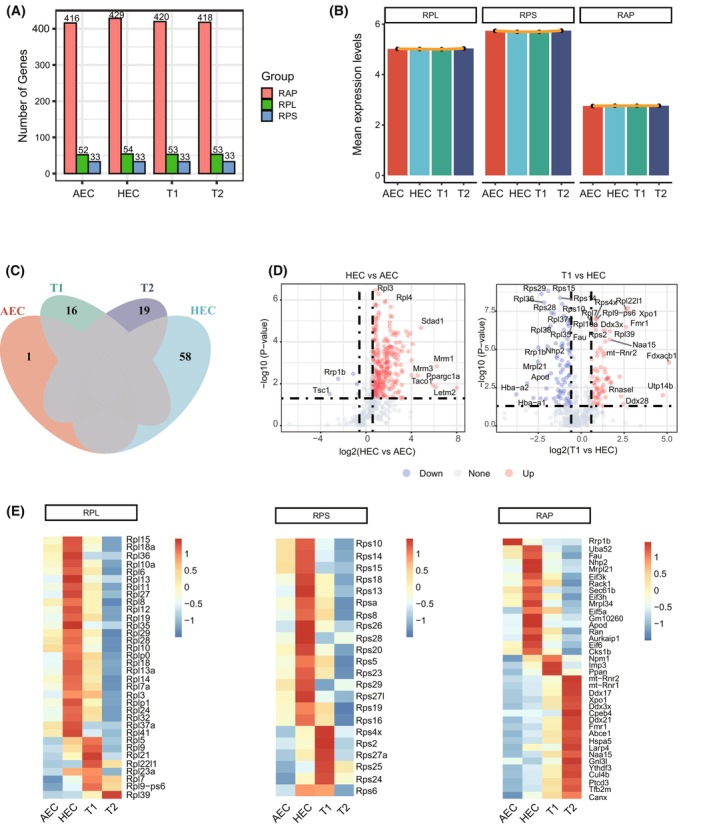
The dynamic changes of ribosomal genes during EHT at the transcriptome level. (A) The distribution and quantity of RPL (large ribosomal subunit protein), RPS (small ribosomal subunit protein) and RAP (ribosome‐associated protein) genes were analyzed using single‐cell transcriptome data during EHT (AEC, *n* = 24; HEC, *n* = 24; T1 and T2 pre‐HSC, *n* = 28 and *n* = 22; all single‐cell data were integrated for analysis). (B) The mean expression level of RPL, RPS and RAP genes during EHT. (C) The stage‐specific ribosome‐related genes during EHT. (D) The differentially expressed ribosome‐related genes in HECs compared to AECs and T1 pre‐HSCs. Data are analyzed by the Wilcoxon rank sum test. (E) Unique dynamic pattern of RP and RAP heterogeneity during EHT. The relative fold change of average gene expression level was represented between adjacent populations.

To precise resolve ribosome heterogeneity, we first employed four datasets from our previous published single‐cell transcriptome data [22], which covered major developmental stages during EHT (AEC, HEC, T1 and T2 pre‐HSC). We then extracted a total of 529 ribosome‐related genes and divided them into three major groups (RPL, RPS and RAP). The distribution and quantity of RPL, RPS and RAP genes were almost similar in the four cell populations, with the most abundant number of RAP genes and the lowest number of RPS genes (Fig. 1A). Notably, opposite expression levels were observed, with the highest expression of RPS genes and the lowest expression of RAP genes, suggesting the active transcription of core RPs than RAP during EHT (Fig. 1B). We also noted that, although the majority of ribosome‐related genes are invariable across EHT, stage‐specific genes were still detected in individual cell populations (Fig. 1C). Especially in HECs, there were more heterogeneous ribosome‐related genes compared to AECs and T1 pre‐HSCs (Fig. 1D). Interestingly, an unique dynamic pattern of RP and RAP heterogeneity was also observed during EHT. HECs expressed more core RPs (e.g. Rps15, Rpl10a and Rplp0), whereas T2 pre‐HSCs expressed more translation‐related RAPs (e.g. Ddx17, Ddx21 and Ddx3x) than other cell populations (Fig. 1E). Together, these results indicated that the formation and potential function of ribosome heterogeneity are continuously reshaped at different times of EHT.

### The expression pattern of ribosomal proteins during EHT at the proteome level

**Fig. 2:**
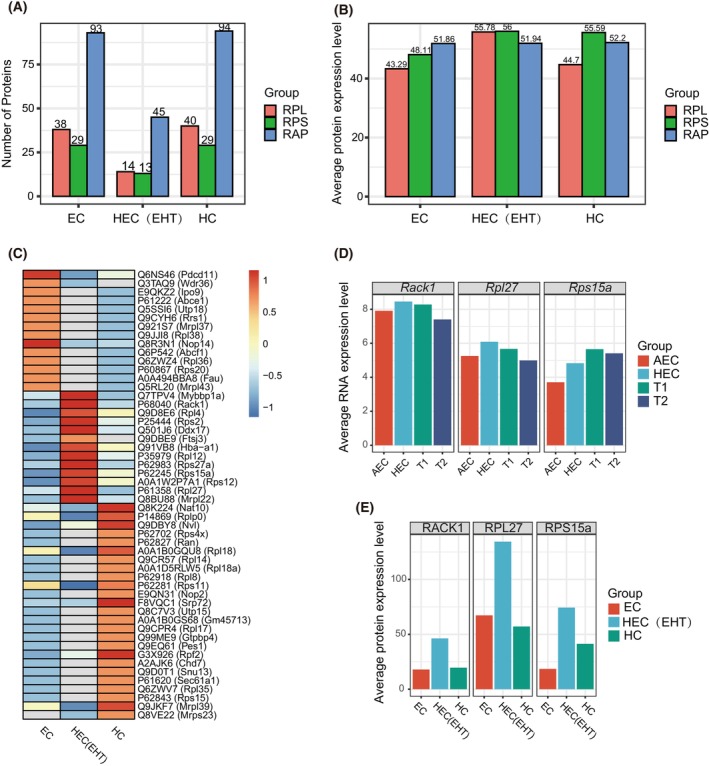
The expression pattern of ribosomal proteins during EHT at the proteome level. (A) The distribution and quantity of RPL, RPS and RAP proteins were analyzed using bulk‐cell proteomic data during EHT (EC: endothelial cell, *n* = 3; HEC: hemogenic endothelial cell, *n* = 3; HC: hematopoietic cell, *n* = 3). (B) The average expression level of RPL, RPS and RAP proteins during EHT. (C) The stage‐specific ribosome‐related proteins during EHT. The relative fold change of average protein expression level was represented between adjacent populations. (D, E) The differentially expressed pattern of RPS15a, RPL27 and RACK1 at transcriptome (D) and proteome (E) levels.

We had observed ribosome heterogeneity at the transcriptome level, whereas there was actually a poor correlation between mRNA expression and protein level during key developmental time points [2]. To further confirm this observation, we reanalyzed the published proteomic data covering three major groups during EHT: endothelial cells (ECs) (CD31^+^ Kit^−^), HECs (CD31^+^ Kit^+^) and HCs (CD31^−^ Kit^+^) [23]. The phenotypic HEC in the public study represented a rough cell population mainly containing the intermediators of EHT, including bone‐fide HECs, pre‐HSCs and several hematopoietic cells, and so we redefined it as HEC (EHT). Consistently, the distribution of RPL, RPS and RAP proteins was almost similar in the three cell populations (Fig. 2A). Nevertheless, the number of RAP proteins was dramatically reduced compared to mRNA expression level, suggesting that this part of RAP might be sufficient to execute the regulatory function (Fig. 2A). We also noted that few ribosomal proteins were detected in HEC (EHT), which may be partly a result of poor proteomic quality. Intriguingly, the expression of RPL, RPS and RAP was equivalent at the protein level (Fig. 2B). Noteworthy, although few stage‐specific ribosome‐related genes were detected in HEC (EHT) (Fig. 2C), we found that several of them (e.g. RPS15a, RPL27 and RACK1) showed upregulated expression both at the transcriptome and proteome level (Fig. 2D,E), suggesting that the upregulation of these genes may finally contribute to the generation of cell‐stage specific heterogeneous ribosomes, which might tend toward to the translation of genes specializing in hematogenic fate during EHT.

### Quantitative analysis of ribosomal protein expression by immunofluorescence

**Fig. 3:**
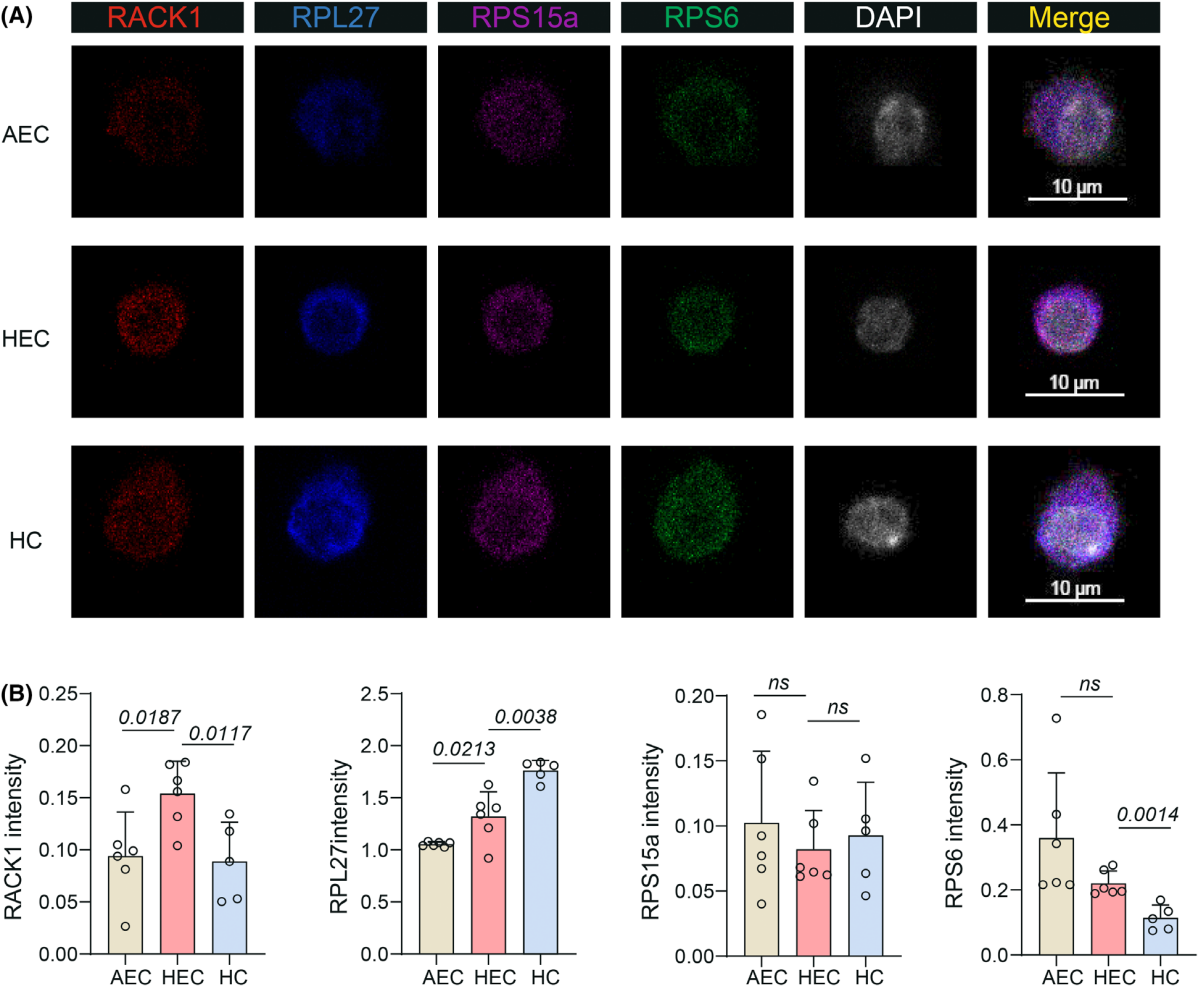
Quantitative analysis of ribosomal protein expression by immunofluorescence. (A) Representative immunofluorescence images showing subcellular localization of RACK1, RPL27, RPS15a and RPS6 in AECs (CD41^−^ CD43^−^ CD45^−^ CD31^+^ CD201^−^ Kit^−^ CD44^+^), HECs (CD41^−^ CD43^−^ CD45^−^ CD31^+^ CD201^+^ Kit^+^ CD44^+^) and HCs (CD45^+^ Kit^+^). Scale bars = 10 μm. (B) Quantitative analysis of ribosomal protein expression through immunofluorescence signal intensity measurement. Data were collected from single AEC (*n* = 6), HEC (*n* = 6) and HC (*n* = 5). Data are presented as the mean ± SD and analyzed by unpaired two‐tailed Student's *t*‐test. Data were collected from three independent experiments.

To further verify our findings in the transcriptome and proteome analysis, we simultaneously sorted individual precisely phenotypic AEC, HEC and HC, and performed fluorescence staining. Under the same experimental conditions and microscope scanning exposure intensity, we first found that the signal of RACK1, RPS15a and RPL27 was overlapped with that of RPS6 (one core component of ribosome), indicating the localization of ribosome (Fig. 3A). Then, through quantitative analysis of the average fluorescence intensity, we found that the expression intensity of RACK1 in HECs was higher than that in AECs and HCs, whereas the expression intensity of RPL27 continued to increase during EHT (Fig. 3B). However, no significant differences were observed in RPS15a and RPS6 (Fig. 3B). Taken together, these results were partially consistent with our findings in the transcriptome and proteome analysis, indicating that the high expression of RACK1 and RPL27 could incorporate into ribosomes and contribute to ribosomal heterogeneity.

### Position of variable ribosome‐related genes within the ribosome during EHT

**Fig. 4:**
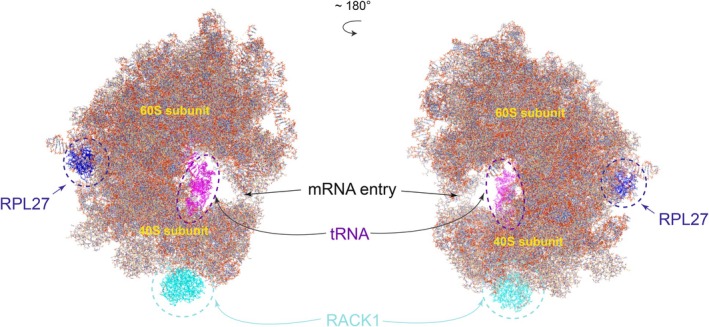
Position of variable ribosome‐related genes within the ribosome during EHT. Visualization of RPL27 and RACK1 in mouse ribosome (PDB: 7cpu). Individual proteins are indicated by different colors.

Further investigation revealed that RACK1 was involved in the initiation of the ribosome quality control [24], whereas RPL27 mutations were commonly found in Diamond–Blackfan anemia [25], suggesting their potential regulatory function in hematopoiesis. To further explore their regulatory mechanism, we used chimerax software and the PDB database (7cpu Mouse ribosome) to analyze the localization of these proteins within the quaternary structure of the mouse ribosome, reflecting how they regulate the ribosome's function. The results showed that RPL27 and RACK1 all appeared to be present at the periphery of the ribosome. We also found RACK1 positioned at the bottom of 40S small ribosomal subunit in the spatial structure (Fig. 4). Interestingly, further investigation revealed that RACK1 could function as a scaffold protein and associate with the 40S small ribosomal subunit to efficiently recruit eukaryotic initiation factor 4E. Otherwise, RACK1 also acted as a ribosomal signaling hub to open binding sites for translational regulators, as well as to regulate mammalian ribosome‐associated quality control by mediating regulatory 40S ribosomal ubiquitylation [6, 26, 27]. Taken together, we hypothesize that binding of these proteins to the surface of the ribosome might be attuned to actively alter the structure or translation of the ribosome by diversity functions.

## Discussion

Ribosome heterogeneity has been confirmed to play an important role in embryonic development. In the present study, we revealed the landscape of ribosome heterogeneity in the embryonic hematopoiesis for the first time. By deeply distinguishing the RPs and RAPs, we preliminarily revealed how ribosome heterogeneity was formed during EHT both at the transcriptome and proteome levels. Although large differences in ribosomal gene expression were found between RNA and protein levels, a conserved pattern still exists during EHT. We found that RPL27 and RACK1 are significantly upregulated both at transcriptome and proteome levels and, structurally, they all exist on the surface of the ribosomes, suggesting that they may bind quickly to the surface of specific ribosomes to change their structure, mRNA reorganization or translation efficiency.

EHT is a complex and highly coordinated process, involving HEC specialization, as well as morphological changes to and proliferation of hematopoietic cells, in an extremely short time window. Notably, our previous study has revealed the dynamic changing of translation efficiency during EHT, showing higher translation rates in HECs when compared to that of AECs and T1 pre‐HSCs [22]. In this study, we further revealed that high ribosome biogenesis activity was accompanied by stage‐specific ribosome gene expression during EHT. Therefore, we speculate that heterogeneous ribosomes might be appropriately formed within the cells and tend to mediate the translation efficiency of specialized genes at different stages of the EHT process, exerting a rapid regulatory role.

At present, the main method for revealing ribosome heterogeneity still relies on high‐precision proteomics technology. Because of the small number of cells during EHT, it is still difficult to guarantee the quality of proteomics. The development of reliable single‐cell proteomics technology in the future will help with further analyses of ribosome heterogeneity in embryonic hematopoiesis.

## Conflicts of interest

The authors declare that they have no conflicts of interest.

## Peer review

The peer review history for this article is available at https://www.webofscience.com/api/gateway/wos/peer‐review/10.1002/2211‐5463.70078.

## Author contributions

BL, YL and JZ conceived and designed the project. DL performed bioinformatics analysis with the help from XT and JZ. XT performed immunofluorescence experiments with the help from JZ. XT and DL analyzed and interpreted the data. XT and JZ wrote the manuscript. All authors reviewed the manuscript.

## Data Availability

The single‐cell transcriptome sequencing data that support the findings of this study are openly available in NCBI's Gene Expression Omnibus and are accessible through: https://www.ncbi.nlm.nih.gov/geo/query/acc.cgi?acc=GSE205595 and https://www.ncbi.nlm.nih.gov/geo/query/acc.cgi?acc=GSE67120; GEO Series accession numbers (GSE185555 and GSE67120). The mass spectrometry proteomics data that support the findings of this study have been deposited in the ProteomeXchange Consortium (http://proteomecentral.proteomexchange.org) via the PRIDE partner repository with the dataset identifier: PXD042649. The structural data that support these findings are openly available in the wwPDB at: https://doi.org/10.2210/pdb7cpu/pdb.
